# Clinical and stool microbiome correlates of simple post-ERCP hyperamylasemia in children undergoing therapeutic ERCP for pancreatobiliary obstructive disorders: an exploratory pilot study

**DOI:** 10.3389/fped.2026.1851821

**Published:** 2026-06-24

**Authors:** Xueqi Wang, Yifan Zhang, Mao Ye, Chihuan Kong, Mei Diao

**Affiliations:** Department of General Surgery, Capital Center for Children’s Health, Capital Medical University, Capital Institute of Pediatrics, Beijing, China

**Keywords:** children, ERCP, hyperamylasemia, microbiome, pancreatobiliary obstruction, pilot study

## Abstract

**Background:**

Simple post-ERCP hyperamylasemia is a common biochemical finding after therapeutic endoscopic retrograde cholangiopancreatography (ERCP), but pediatric data integrating procedural characteristics with stool microbiome features remain limited.

**Methods:**

We performed an exploratory single-center observational pilot study of 24 successful therapeutic ERCP procedures in children younger than 18 years with pancreatobiliary obstructive disorders between January 2024 and December 2025. The primary endpoint was simple post-ERCP hyperamylasemia, defined as serum amylase >3 times the upper limit of normal within 24 h after ERCP without new or worsening abdominal pain. Baseline clinical variables, predefined stool microbiome features derived from pre-ERCP metagenomic data (Shannon diversity, *Enterococcus* abundance, and *Bifidobacterium* abundance), and intraprocedural variables were compared between groups. Exploratory signal prioritization was used only to identify candidate associations for future validation.

**Results:**

Hyperamylasemia occurred in 8/24 procedures (33.3%). Compared with non- hyperamylasemia group, the affected children had higher baseline gamma-glutamyl transferase and C-reactive protein, longer procedure time, more difficult cannulation, more inadvertent pancreatic duct cannulation, more pancreatic contrast injection, and more rescue precut access. Stool microbiome features in the hyperamylasemia group included lower Shannon diversity, higher *Enterococcus* abundance, and lower *Bifidobacterium* abundance. Procedure time and Shannon diversity emerged as the most interpretable combined signals, but all model estimates should be viewed cautiously because of the small event count.

**Conclusion:**

In this pilot dataset, simple post-ERCP hyperamylasemia clustered with technically demanding procedures and a low-diversity, *Enterococcus*-enriched stool microbiome profile. These findings are hypothesis-generating and require prospective multicenter validation before they can inform pediatric ERCP surveillance or risk-stratification research.

## 1 Introduction

Children with chronic pancreatitis and other pancreatobiliary obstructive disorders may present with recurrent pain, nutritional compromise, impaired quality of life, and substantial genetic or structural disease burden (1–6). Endoscopic retrograde cholangiopancreatography (ERCP) is a fluoroscopy-guided endoscopic procedure used to access the biliary and pancreatic ducts for diagnostic assessment and therapeutic intervention. And as the diagnostic and therapeutic role of endoscopy has expanded, ERCP has become an important option for ductal decompression, stone extraction, stenting, and targeted intervention in selected pediatric patients (7–12). Although pediatric ERCP is generally feasible and safe in experienced centers, adverse events remain clinically important because pediatric evidence is still derived largely from referral-center series and collaborative cohorts rather than large prospective risk-modeling studies (8–12).

Hyperamylasemia refers to an increase in serum amylase above the laboratory upper limit of normal; after ERCP, a marked enzyme rise may reflect transient pancreatic irritation even when the clinical criteria for post-ERCP pancreatitis are not met. Among post-ERCP adverse events, simple hyperamylasemia occupies a practical grey zone: it is more frequent than clinically overt post-ERCP pancreatitis and may trigger additional biochemical monitoring or prolonged observation even without progressive symptoms. Its interpretation therefore requires integration of symptoms, procedural details, and standardized adverse-event definitions (13–23). Adult and mixed-age studies consistently identify difficult cannulation, repeated pancreatic duct access, pancreatic contrast injection, and prolonged procedures as major determinants of pancreatic irritation after ERCP (14–21, 23). However, pediatric evidence remains comparatively limited and is still dominated by descriptive series rather than integrated analyses of procedural and biological susceptibility (8–12).

Concurrently, microbiome studies in pediatric chronic pancreatitis and acute pancreatitis suggest that gut dysbiosis may accompany inflammatory susceptibility (24–27). Whether stool microbiome features are associated with simple post-ERCP hyperamylasemia in children remains uncertain. We selected simple hyperamylasemia rather than clinically overt post-ERCP pancreatitis because overt pediatric PEP was not frequent enough in this pilot cohort for meaningful analysis, whereas simple hyperamylasemia provided a standardized early biochemical endpoint for studying pancreatic irritation after therapeutic ERCP. We therefore conducted a basic pilot study to explore the clinical and stool microbiome correlates of this endpoint in children with pancreatobiliary obstructive disorders, rather than to develop or validate a clinical prediction model.

## 2 Materials and methods

### 2.1 Study design and setting

This was a single-center exploratory observational pilot study conducted at Capital Center for Children's Health, Capital Medical University. Twenty-four successful therapeutic ERCP procedures from children younger than 18 years with pancreatobiliary obstructive disorders were included in the analytic dataset, with one procedure contributed by each child. The study was designed to evaluate associations and prioritize candidate signals rather than to develop a definitive clinical prediction tool.

### 2.2 Patient recruitment and eligibility criteria

During the study period from January 2024 to December 2025, 28 pediatric ERCP procedures were screened. No purely diagnostic ERCP procedures were performed during this interval; all screened procedures were therapeutic ERCPs. Inclusion criteria were confirmed pancreatobiliary obstructive disease, age <18 years, fulfillment of ERCP indications, availability of peri-procedural laboratory testing, and availability of a pre-procedural stool sample for exploratory metagenomic analysis. Exclusion criteria were incomplete records, failed ERCP, concurrent acute pancreatitis at baseline that could confound endpoint assessment, missing 24-hour serum amylase measurements, and repeated procedures from the same child beyond the index procedure selected for this analysis.

### 2.3 Peri-procedural management and ERCP technique

Before ERCP, all children were undergone routine laboratory evaluation, imaging review, and anesthetic assessment. ERCP was performed using a Fuji duodenoscope ED-530XT with guidewire-assisted cannulation. Therapeutic maneuvers included sphincterotomy, balloon dilation, stent placement, stone extraction, and related interventions according to cholangiopancreatographic findings. Peri-procedural prophylaxis, hydration, and post-procedural supportive care followed routine institutional practice.

Difficult cannulation was defined as failure to achieve the intended duct access within 5 min after papillary visualization, more than five cannulation attempts, or repeated unintended pancreatic duct access requiring a change in access strategy. Inadvertent pancreatic duct cannulation was defined as unplanned guidewire or catheter entry into the pancreatic duct before intended target-duct access. Pancreatic contrast injection was recorded when contrast was fluoroscopically visualized in the pancreatic duct. Rescue precut access was defined as the use of needle-knife fistulotomy, transpancreatic septotomy, or a related rescue access technique after standard cannulation was unsuccessful or repeated pancreatic access occurred. When standard biliary access failed or repeated pancreatic duct access occurred, rescue techniques were used according to predefined indications and endoscopist judgment.

### 2.4 Outcome definitions

The primary endpoint was simple post-ERCP hyperamylasemia, defined as serum amylase >3 times the upper limit of normal within 24 h after ERCP in the absence of additional or worsening abdominal pain (20–22). Children meeting criteria for clinically overt post-ERCP pancreatitis were not assigned to the endpoint group. Serum amylase levels and numerical rating scale (NRS) pain scores were recorded before ERCP and at 2, 24, 48, and 72 h after the procedure respectively.

### 2.5 Clinical covariates

Baseline variables included age, sex, body weight, abdominal pain pattern, history of recurrent pancreatitis, prior biliary or pancreatic interventions, anatomical anomalies, laboratory indices (white blood cell count, C-reactive protein, bilirubin, alanine aminotransferase, aspartate aminotransferase, gamma-glutamyl transferase, albumin, lipase, and baseline amylase), and imaging characteristics. Procedural variables included difficult cannulation, inadvertent pancreatic duct cannulation, pancreatic contrast injection, rescue precut access, prophylactic pancreatic stenting, and total procedure time. Peri-procedural supportive and preventive care followed contemporary ERCP safety guidance and routine institutional practice (16–21, 28–31).

### 2.6 Stool metagenomic feature derivation

Pre-ERCP fecal specimens were collected within 12 h before ERCP. All samples were obtained before bowel preparation and before peri-procedural antibiotics (24/24, 100%). All specimens were stored at −80 °C until processing.

Total genomic DNA was extracted from fecal samples using the DNeasy Blood & Tissue Kit (QIAGEN, Redwood City, CA, USA) according to the manufacturer's instructions. Shotgun metagenomic sequencing was performed on the Illumina NovaSeq 6000 platform using paired-end 150-bp reads. Each sample yielded approximately 6–10 Gb of raw sequencing data, corresponding to about 40–67 million raw reads. After quality control, the clean-read retention rate was approximately 92.5%, corresponding to about 37–62 million clean reads per sample. Quality assessment and processing, including read filtering and host-read removal, used FastQC, MultiQC, fastp, and Bowtie2. Assembly, gene prediction, clustering, taxonomic or functional annotation, and downstream microbiome analysis used MEGAHIT, QUAST, MetaGeneMark, CD-HIT, DIAMOND, MEGAN, EggNOG-mapper, DeepARG, MinPath, and R/vegan. Reference databases used for the analyses reported here included NR and KEGG. Abundance estimates were normalized by correcting read-mapping counts for gene length and sequencing depth; downstream group comparisons used relative or standardized abundance values aggregated to taxonomic or functional levels.

Because this pilot report was designed to evaluate a small, biologically interpretable signal set rather than perform broad discovery analysis, it is mainly focused on three prespecified summary features derived from stool microbiome data: Shannon diversity, *Enterococcus* genus-level relative abundance, and *Bifidobacterium* genus-level relative abundance. Shannon diversity was calculated from normalized genus-level relative-abundance profiles using the vegan package in R. *Enterococcus* and *Bifidobacterium* relative abundances were calculated at the genus level. These features were selected *a priori* to reduce dimensionality and multiple testing in a very small cohort and because prior pediatric and translational studies suggest that dysbiosis, *Enterococcus* enrichment, and *Bifidobacterium* depletion may track pancreatitis susceptibility or severity (24–27, 32).

### 2.7 Statistical analysis

Continuous variables are summarized as mean ± standard deviation and categorical variables as number (percentage). Between-group comparisons were performed using Welch t tests for continuous variables and Fisher exact tests for categorical variables. Clinical data analyses were performed primarily with IBM SPSS Statistics version 26.0 (IBM Corp., Armonk, NY, USA). Metagenomic diversity analysis and visualization were performed primarily with R version 4.2.2 (R Foundation for Statistical Computing, Vienna, Austria) and relevant packages, including vegan and VennDiagram. No formal sample-size calculation was performed because the cohort reflected the number of eligible procedures during the study period. Given the pilot design and low event count, all inferential analyses were intended to describe associations and prioritize candidate signals rather than estimate definitive multivariable effects.

Candidate features were explored using penalized logistic regression after z-score standardization, with complementary random-forest importance ranking used only as a descriptive robustness check. To avoid presenting an overfitted multivariable prediction model, the final analysis was reframed as a two-feature exploratory signal combining one procedural feature and one microbiome feature selected on the basis of biological interpretability, collinearity, and internal discrimination. Apparent area under the receiver operating characteristic curve (AUC), leave-one-out AUC, Brier score, and an optimism-corrected C-index estimated from 200 bootstrap resamples are reported only as internal descriptive metrics. They should not be interpreted as evidence of a validated or deployable clinical prediction model.

### 2.8 Ethics statement

The study was approved by the Institutional Review Board of Capital Institute of Pediatrics (Approval No. SHERLL2024053). Written informed consent was obtained from the parents or legal guardians of all participants, with assent from children when appropriate. The study was conducted in accordance with the Declaration of Helsinki.

## 3 Results

### 3.1 Patient flow and cohort overview

**Figure 1:**
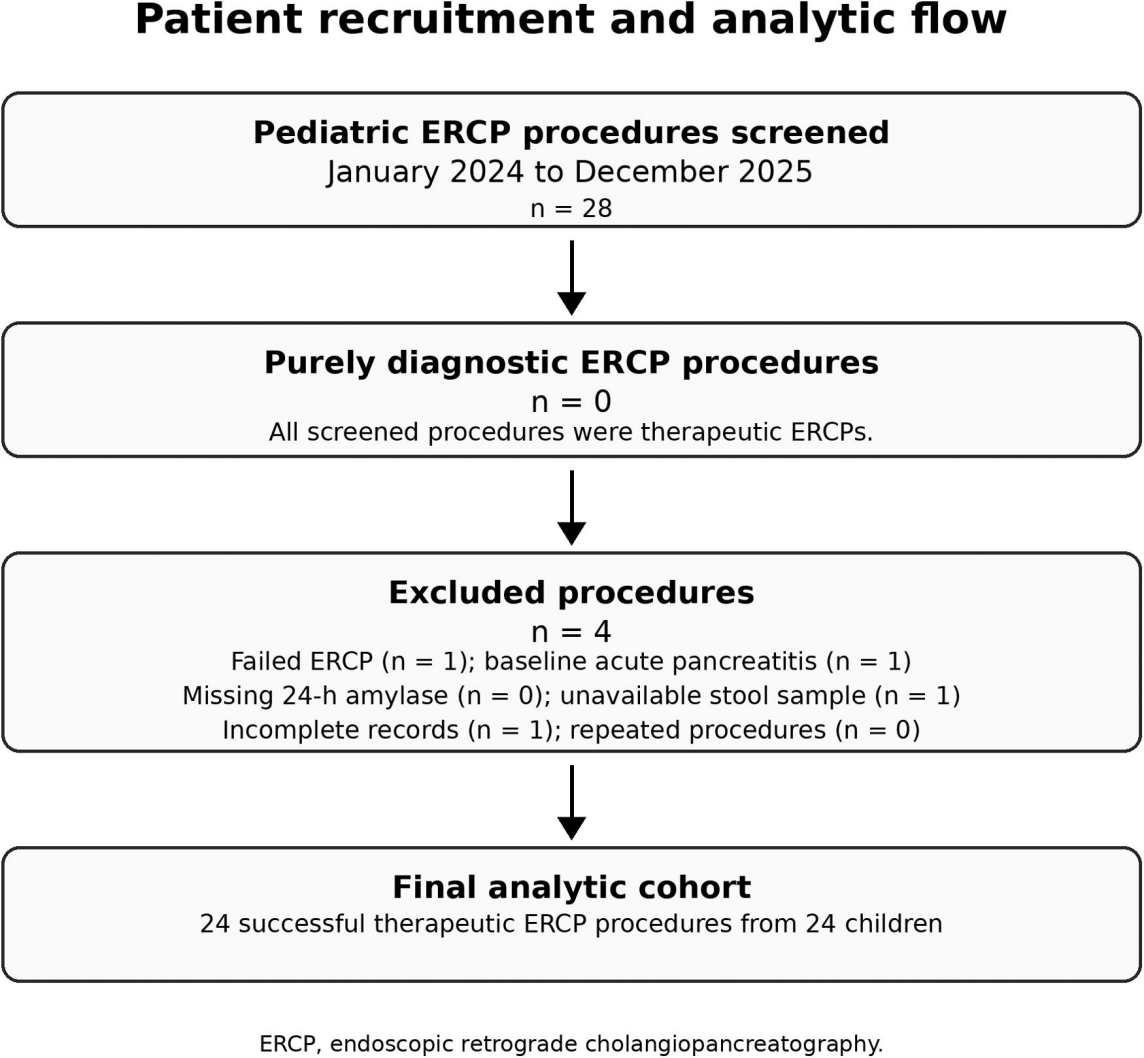
Patient recruitment and analytic flow. Twenty-eight pediatric ERCP procedures were screened. No purely diagnostic procedures were performed. Four procedures were excluded: failed ERCP (*n* = 1), baseline acute pancreatitis (*n* = 1), unavailable pre-procedural stool sample (*n* = 1), and incomplete records (*n* = 1). The final analytic cohort included 24 successful therapeutic ERCP procedures from 24 children. ERCP, endoscopic retrograde cholangiopancreatography.

A patient-flow summary is provided in Figure 1. In brief, 28 pediatric ERCP procedures were screened during the study period. No purely diagnostic ERCPs were performed. After exclusion of four procedures (failed ERCP, *n* = 1; concurrent acute pancreatitis at baseline, *n* = 1; unavailable pre-procedural stool sample, *n* = 1; incomplete records, *n* = 1), 24 successful therapeutic ERCP procedures from 24 children were included in the analytic cohort. No procedures were excluded for missing 24-h amylase measurement or repeated procedures.

The cohort comprised 24 children undergoing 24 analyzed ERCP procedures (mean age 11.8 ± 2.4 years; 14/24 girls). A history of recurrent pancreatitis was present in 9 children (37.5%), and a ductal/anatomic anomaly was present in 5 (20.8%). Simple post-ERCP hyperamylasemia occurred in 8/24 procedures (33.3%). The present analysis focused on this predefined biochemical endpoint and not on modeling clinically overt post-ERCP pancreatitis.

Baseline demographic differences between the two groups were modest. Nonetheless, the hyperamylasemia group had higher baseline GGT and CRP, lower microbial alpha diversity, higher *Enterococcus* abundance, and lower *Bifidobacterium* abundance (Table 1).

**Table 1:** Baseline clinical and stool microbiome characteristics.

Variables	Overall (*n* = 24)	Hyperamylasemia (*n* = 8)	No hyperamylasemia (*n* = 16)	*p* value
Age, years	11.8 ± 2.4	12.4 ± 2.6	11.5 ± 2.3	0.418
Sex, female/male	14/24 (58.3%)	5/8 (62.5%)	9/16 (56.2%)	1.000
History of recurrent pancreatitis	9/24 (37.5%)	5/8 (62.5%)	4/16 (25.0%)	0.099
Pancreatic duct/anatomic anomaly	5/24 (20.8%)	3/8 (37.5%)	2/16 (12.5%)	0.289
Baseline GGT, U/L	129.0 ± 59.3	197.0 ± 43.3	95.0 ± 28.5	<0.001
Baseline CRP, mg/L	6.8 ± 4.2	11.1 ± 3.9	4.7 ± 2.2	0.002
Baseline total bilirubin, umol/L	19.7 ± 5.2	18.8 ± 5.4	20.1 ± 5.2	0.586
Shannon diversity index	3.29 ± 0.41	2.86 ± 0.30	3.50 ± 0.26	<0.001
*Enterococcus* relative abundance, %	5.61 ± 2.09	7.88 ± 1.61	4.47 ± 1.19	<0.001
*Bifidobacterium* relative abundance, %	4.10 ± 2.27	1.46 ± 0.50	5.41 ± 1.49	<0.001

Values are presented as mean ± standard deviation or number (%).

### 3.2 Procedural characteristics and short-term trajectories

Children who developed simple hyperamylasemia underwent more technically demanding procedures. Difficult cannulation was observed in 6/8 (75.0%) endpoint-positive procedures versus 4/16 (25.0%) controls; inadvertent pancreatic duct cannulation occurred in 75.0% vs. 18.8%, and pancreatic contrast injection in 62.5% vs. 12.5%. Procedure time was longer by nearly 18 min on average in the endpoint-positive group (Table 2).

**Table 2:** ERCP-related procedural characteristics.

Variables	Overall (*n* = 24)	Hyperamylasemia (*n* = 8)	No hyperamylasemia (*n* = 16)	*p* value
Procedure time, min	36.3 ± 12.6	48.1 ± 12.8	30.4 ± 7.5	0.005
Difficult cannulation	10/24 (41.7%)	6/8 (75.0%)	4/16 (25.0%)	0.032
Inadvertent pancreatic duct cannulation	9/24 (37.5%)	6/8 (75.0%)	3/16 (18.8%)	0.021
Pancreatic contrast injection	7/24 (29.2%)	5/8 (62.5%)	2/16 (12.5%)	0.021
Rescue precut access	5/24 (20.8%)	4/8 (50.0%)	1/16 (6.2%)	0.028
Prophylactic pancreatic stent	4/24 (16.7%)	1/8 (12.5%)	3/16 (18.8%)	1.000

Serum amylase values peaked at 24 h and then declined over 48–72 h, while pain scores improved over time in both groups. This pattern is consistent with transient biochemical pancreatic irritation rather than clinically progressive post-ERCP pancreatitis (Table 3). By study definition, the hyperamylasemia group had marked enzyme elevation without pain progression beyond baseline.

**Table 3:** Serial serum amylase and pain trajectories after ERCP.

Variables	Overall (*n* = 24)	Hyperamylasemia (*n* = 8)	No hyperamylasemia (*n* = 16)	*p* value
Serum amylase before ERCP, U/L	80.1 ± 15.4	84.0 ± 13.6	78.1 ± 16.3	0.364
Serum amylase 2 h after ERCP, U/L	221.2 ± 51.3	228.4 ± 59.3	217.6 ± 48.5	0.665
Serum amylase 24 h after ERCP, U/L	208.1 ± 157.3	419.5 ± 56.0	102.4 ± 26.8	<0.001
Serum amylase 48 h after ERCP, U/L	141.4 ± 86.3	244.1 ± 70.8	90.1 ± 25.1	<0.001
Serum amylase 72 h after ERCP, U/L	90.2 ± 34.1	130.8 ± 26.7	69.9 ± 11.8	<0.001
Pain NRS before ERCP	6.0 ± 1.0	5.9 ± 0.6	6.1 ± 1.2	0.514
Pain NRS 2 h after ERCP	2.4 ± 1.0	3.1 ± 1.2	2.0 ± 0.6	0.040
Pain NRS 24 h after ERCP	1.7 ± 1.0	2.4 ± 0.9	1.4 ± 1.0	0.026
Pain NRS 48 h after ERCP	1.0 ± 0.6	1.4 ± 0.5	0.8 ± 0.5	0.026
Pain NRS 72 h after ERCP	0.7 ± 0.6	1.0 ± 0.8	0.6 ± 0.5	0.170

### 3.3 Exploratory signal prioritization

Exploratory screening consistently highlighted both inflammatory/procedural variables and stool microbiome features. Penalized regression retained baseline GGT, CRP, Shannon diversity, *Enterococcus*, *Bifidobacterium*, and procedure time, whereas random-forest ranking emphasized *Bifidobacterium* depletion, baseline GGT, *Enterococcus* enrichment, and low Shannon diversity as the strongest non-random signals (Table 4).

**Table 4:** Exploratory feature prioritization outputs and internal model performance.

Metric/retained feature	Result
Baseline GGT	Positive coefficient
Baseline CRP	Positive coefficient (weak)
Shannon diversity	Negative coefficient
Enterococcus abundance	Positive coefficient
Bifidobacterium abundance	Negative coefficient
Procedure time	Positive coefficient
Random-forest top-ranked features	Bifidobacterium, baseline GGT, Enterococcus, Shannon diversity, baseline CRP, procedure time
Exploratory two-feature signal	Procedure time+Shannon diversity
Apparent AUC	0.93
Leave-one-out AUC	0.85
Optimism-corrected C-index (200 bootstraps)	0.91
Brier score	0.091

**Figure 2:**
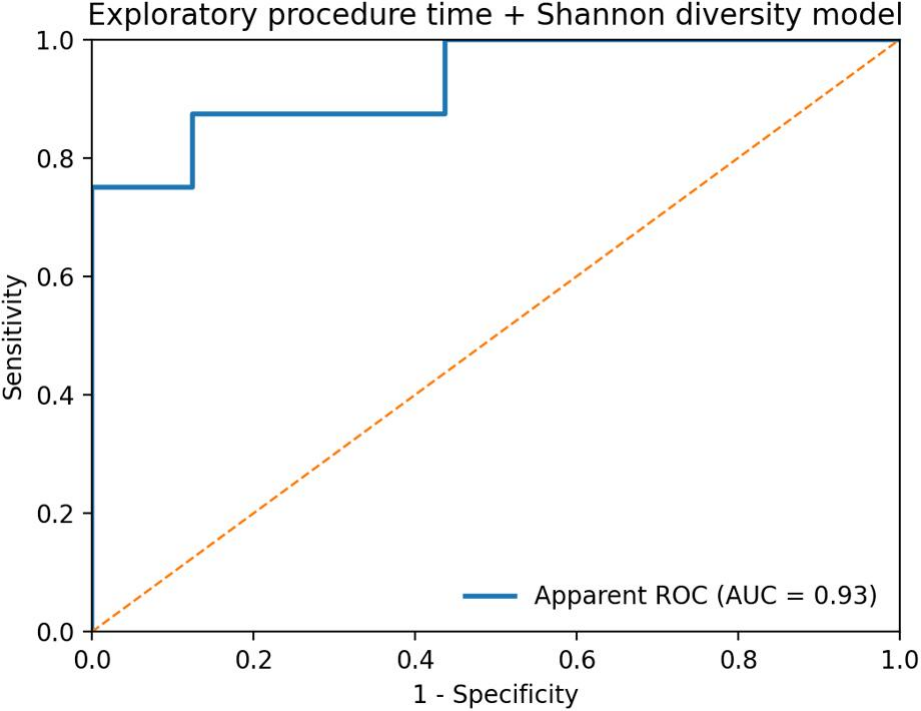
Receiver operating characteristic curve of the internally evaluated exploratory signal combining procedure time and shannon diversity. Internal illustration only: apparent AUC = 0.93, leave-one-out AUC = 0.85, and optimism-corrected C-index = 0.91; these estimates are likely optimistic in a dataset with only eight endpoint events.

Given the limited sample size and collinearity among variables, we avoided claiming a stable prediction model. Instead, procedure time and Shannon diversity were retained as the most interpretable combined procedural-microbiome signal. The apparent and internally corrected discrimination metrics are presented only to illustrate signal separation within this dataset, and should be interpreted as optimism-prone descriptive estimates rather than evidence of predictive robustness (Table 4 and Figure 2).

## 4 Discussion

ERCP, as an effective method, is currently widely used in the diagnosis and treatment of pancreatic and biliary diseases. However, the occurrence of post- ERCP hyperamylasemia is relatively common, which was widely attracted by endoscopic clinicians. This exploratory pilot study suggests that simple hyperamylasemia after pediatric ERCP is not randomly distributed; rather, it clusters around technically demanding cannulation and a pre-existing low-diversity microbial state. The 33.3% event rate should not be interpreted as a pancreatitis rate, because simple biochemical hyperamylasemia is a broader and more frequent endpoint than clinically overt post-ERCP pancreatitis (8–13, 20, 21).

We chose simple post-ERCP hyperamylasemia as the primary endpoint for practical and methodological reasons. Clinically overt pediatric PEP is uncommon, and the number of overt events in a single-center pediatric therapeutic ERCP cohort is usually insufficient for stable association analysis. In contrast, a marked postprocedural amylase elevation is an early, standardized biochemical signal of pancreatic irritation that may prompt repeated laboratory testing, closer observation, delayed feeding decisions, or delayed discharge even when symptoms do not progress. Therefore, the endpoint has practical relevance for postprocedural surveillance, while remaining clearly distinct from clinically overt PEP. We have revised the manuscript to emphasize this distinction and to avoid presenting the endpoint as equivalent to a patient-centered pancreatitis outcome.

The procedural signature in this cohort mirrors the strongest signals from adult and mixed-age ERCP literature. Difficult or prolonged access, repeated pancreatic duct entry, pancreatic contrast injection, and rescue precut access all tracked with the endpoint, supporting the concept that papillary trauma and transient outflow obstruction are important drivers of post-ERCP pancreatic enzyme release (14–21, 33, 34). At the same time, the absence of progressive pain in the endpoint group underscores the clinical distinction between simple hyperamylasemia and true post-ERCP pancreatitis (20, 21).

The stool microbiome findings add a biologically plausible but clearly preliminary layer to these observations. Pediatric acute pancreatitis has been associated with altered alpha and beta diversity and enrichment of *Enterococcus faecalis* in severe disease, while pediatric chronic pancreatitis has also been linked with disordered gut microbial profiles (24, 27). Adult and translational pancreatitis studies further suggest that dysbiosis may interact with intestinal barrier dysfunction, systemic inflammation, microbial translocation, and bile-acid or metabolite signaling (25, 26). In experimental work, *Bifidobacterium* spp. and their metabolite lactate have been reported to inhibit pancreatic and systemic inflammatory responses in acute pancreatitis models (32). These studies provide context for the observed pattern of lower Shannon diversity, higher *Enterococcus* abundance, and lower *Bifidobacterium* abundance, but they do not establish causality in the present cohort.

Several causal explanations remain possible. A low-diversity, *Enterococcus*-enriched stool profile could reflect the underlying pancreatobiliary disorder, biliary stasis, prior hospitalization, medication exposure, diet, or inflammatory burden rather than intrinsic susceptibility to post-ERCP pancreatic irritation. Conversely, a dysbiotic state might lower the threshold for enzyme leakage after papillary trauma through altered mucosal barrier function, immune tone, or microbial metabolites. Because stool was collected before ERCP, the association is temporally compatible with a pre-procedural correlate, but this design cannot establish directionality or exclude residual confounding.

Baseline GGT and CRP also differed markedly between groups. These laboratory signals may reflect a broader phenotype of biliary stasis, inflammatory activation, or more complex anatomy that leads to longer procedures. Related mechanistic work also suggests that GGT1-related signaling may deserve further study in post-ERCP injury (35). In a larger prospective cohort, these variables should be evaluated alongside detailed anatomic descriptors, operator-level factors, hydration strategy, and prophylactic medications (16–19, 28–31).

Clinically, these signals may help identify children who warrant closer peri-procedural surveillance and meticulous prevention: avoidance of unnecessary pancreatic injections, early expert rescue access rather than repeated standard attempts, careful hydration, and pharmacologic prophylaxis when appropriate (17–19, 23, 28–31, 33). However, most prevention evidence derives from adult ERCP cohorts, so pediatric extrapolation should remain cautious until larger child-specific studies are available. The current findings should be used to formulate future multicenter hypotheses rather than to guide individual risk prediction.

### 4.1 Limitations

This study has several limitations. First, the sample size was small, with only eight endpoint-positive procedures, so no stable multivariable inference should be claimed. The LASSO, random-forest ranking, AUC, leave-one-out validation, and bootstrap metrics were used only for exploratory signal prioritization and internal illustration. In a dataset of this size, all performance estimates are highly susceptible to instability, overfitting, and optimism bias.

Second, the single-center design and heterogeneous procedure-level case mix constrain generalizability. Children with chronic pancreatitis, recurrent pancreatitis, biliary obstruction, and anatomic anomalies may differ in baseline inflammation, stool microbiome composition, procedural difficulty, and enzyme response. The observed microbiome associations may therefore partly reflect underlying disease biology rather than susceptibility to postprocedural pancreatic irritation. Sensitivity analyses excluding chronic pancreatitis or major anatomic anomalies were not performed because the resulting subgroup and event counts would be too small for interpretable inference.

Third, several variables that can influence stool microbiome composition or post-ERCP outcomes were not fully controlled, including prior antibiotic exposure, probiotic use, proton pump inhibitor therapy, dietary variation, bowel preparation, peri-procedural hydration, prophylactic nonsteroidal anti-inflammatory drug use, and previous hospitalization history. Fourth, the stool microbiome analysis was intentionally restricted to a small set of prespecified summary features and should not be interpreted as comprehensive microbiome profiling. This restricted scope reduced dimensionality but may have overlooked other relevant taxa, functional pathways, or metabolomic signatures. Finally, the internal two-feature signal was evaluated only within the derivation dataset and should be regarded strictly as hypothesis-generating.

### 4.2 Conclusion

In this exploratory pilot dataset, simple post-ERCP hyperamylasemia was associated with technically demanding procedures, higher inflammatory markers, and stool microbiome features characterized by reduced diversity, *Enterococcus* enrichment, and *Bifidobacterium* depletion. The combined clinical-microbiome signal is biologically interesting but requires prospective external validation before it can inform pediatric ERCP surveillance or future risk-stratification research.

## Data Availability

The original contributions presented in the study are included in the article/Supplementary Material, further inquiries can be directed to the corresponding authors.
